# Quantitative Genomic Dissection of Soybean Yield Components

**DOI:** 10.1534/g3.119.400896

**Published:** 2019-12-09

**Authors:** Alencar Xavier, Katy M. Rainey

**Affiliations:** * Department of Agronomy, Purdue University, West Lafayette IN 47907 and; † Department of Biostatistics, Corteva Agrisciences, Johnston IA 50131

**Keywords:** soybean, genomic prediction, GWAS, GxE, yield, yield components, heritability, SoyNAM

## Abstract

Soybean is a crop of major economic importance with low rates of genetic gains for grain yield compared to other field crops. A deeper understanding of the genetic architecture of yield components may enable better ways to tackle the breeding challenges. Key yield components include the total number of pods, nodes and the ratio pods per node. We evaluated the SoyNAM population, containing approximately 5600 lines from 40 biparental families that share a common parent, in 6 environments distributed across 3 years. The study indicates that the yield components under evaluation have low heritability, a reasonable amount of epistatic control, and partially oligogenic architecture: 18 quantitative trait loci were identified across the three yield components using multi-approach signal detection. Genetic correlation between yield and yield components was highly variable from family-to-family, ranging from -0.2 to 0.5. The genotype-by-environment correlation of yield components ranged from -0.1 to 0.4 within families. The number of pods can be utilized for indirect selection of yield. The selection of soybean for enhanced yield components can be successfully performed via genomic prediction, but the challenging data collections necessary to recalibrate models over time makes the introgression of QTL a potentially more feasible breeding strategy. The genomic prediction of yield components was relatively accurate across families, but less accurate predictions were obtained from within family predictions and predicting families not observed included in the calibration set.

Soybean is a field crop of major importance due to its seed composition, containing approximately 40% protein and 20% oil. Its unique composition and scalable production make soy a key crop to world-wide food security (Qiu *et al.* 2013). However, soybean germplasm has narrow genetic basis (Carter *et al.* 2004, Mikel *et al.* 2010) that has limited the rate of genetic gains of yield grain to 29 kg/ha/year in North America (Rincker *et al.* 2014). Better breeding strategies are needed to explore soybeans’ full genetic potential (Specht *et al.* 1999, 2014), and a possible approach to increase grain yield is through trait dissection, breaking down yield into yield components. In fact, whereas modern cultivars have around 30 pods per plant (Kahlon *et al.* 2011), some accessions have as many as 200 pods per plant (Zhang *et al.* 2015).

Kahlon and Board (2012) contrasted cultivars released over the past few decades and observed that grain yield increases may have been triggered by changes in yield components over time, particularly in pods and nodes. Suhre *et al.* (2014) found that the number of nodes and pods per node have steadily increased in cultivars released from 1920 to 2010. The number of pods and nodes are key yield-driver (Robinson *et al.* 2009) that reflects the efficiency of the complex physiological process (Board and Tan 1995). These yield components can be increased at farming levels with good agronomic practices and high-end genetics (Board and Kahlon 2011, Kahlon *et al.* 2011). However, the labor-intensive phenotyping of counting soybean pods and nodes can restrict the number of entries and most studies have been conducted with a small number of genotypes (Egli and Bruening 2006, Robinson *et al.* 2009, Kahlon *et al.* 2011, Nico *et al.* 2019).

The first large-scale genetic assessment of complex traits was performed in the SoyNAM population, where 5600 genotypes from 40 bi-parental families sharing a common parent were phenotyped for various agronomic traits (Xavier *et al.* 2016, Xavier *et al.* 2017a, Diers *et al.* 2018). Whereas soybeans have constrained genetic diversity (Carter *et al.* 2004), the SoyNAM is a relatively rich panel of locally adapted genotypes that represents an invaluable resource for the breeding community.

From a preliminary analysis in the SoyNAM population, Xavier *et al.* (2017a) found that grain yield presents strong genetic correlation to yield components, canopy development, and the length of the reproductive period. The latter is a function of days to flowering and days to maturity, both traits controlled by a few major genes (Watanabe *et al.* 2009, 2011, Xia *et al.* 2012, Langewisch *et al.* 2014). The genetic architecture of canopy development has been recently described by Xavier *et al.* (2017b) and Kaler *et al.* (2018). However, the in-depth genetic architecture of yield components had not been characterized with sufficient power and resolutions.

This study aims to conduct a set of quantitative genetic analyses performed with genome-wide markers to unravel the underlying architecture of yield components and assess potential breeding applications. Our evaluation approach includes comparing different strategies for genomic prediction within and across family; perform genomic covariance analysis to uncover the pleiotropy between yield and yield components, as well as the amount of genetic variation attributed to epistasis and genotype-by-environment interactions; and multi-approach association studies to identify regions containing QTL with the potential to be deployed for marker-assisted selection.

## Methods

### Population

The panel under evaluation is a nested association panel, namely the SoyNAM populations, where the standard parent IA3023 (Dairyland DSR365 x Pioneer P9381) was crossed to 40 founder parents that attempt to capture the diversity of public germplasm, each family comprising approximately 140 individuals. Among the 40 founder parents, 17 lines are U.S. elite public germplasm, 15 have diverse ancestry, and eight are planted introductions. The descriptions of parents are available https://www.soybase.org/SoyNAM/. The population’s maturity ranged from late maturity group II to early maturity group IV. More details about the population composition are available in Diers *et al.* (2018) and Xavier *et al.* (2018). After quality control based on segregation patterns, 5363 individuals were used for this study.

### Experimental design

The experiment was conducted under a modified augmented design, with a 7:1 lines-to-check ratio, in two Purdue University research centers: Throckmorton-Purdue Agricultural Center (TPAC) located in Throckmorton, Indiana, and at the Agronomy Center for Research and Education (ACRE) in West Lafayette, Indiana. The experiments were planted during the third week of May in two-row plots (2.9m × 0.76m), at a density of approximately 36 plants m^-2^. The phenotypes were collected in 10 field blocks, these being distributed as 4 adjacent blocks in 2013, 4 adjacent blocks in 2014 and 2 field blocks in different locations in 2015. In 2013 and 2014 the experiments were conducted at the ACRE farm, where each field block contained all 40 families with 35 recombinant inbred lines (RIL) per family, that is, one-quarter of the total number of RILs. In 2013 and 2014 RILs were not replicated, but the same checks were used across fields. In 2015, the experiments were conducted on 6 of the 40 SoyNAM families in two locations, ACRE and TPAC, with two replicates per location.

### Phenotyping

The number of pods and nodes was counted in the main stem, between phenological stages R5 and R7, averaging the counts of 3, 6 and 4 representative plants per plot in 2013, 2014 and 2015 respectively. The variable number of subsamples varied according to the resources available each year. The number of pods per node was obtained by the ratio. Grain yield was collected at harvest, converting the grain weight from individual plots to bushels per acre adjusted to 13% grain moisture. The number of days to maturity (Fehr *et al.* 1971) was collected by scoring the plots every 3 days from the time where the first mature plot was observed, using back-and-forth scoring to assign the plots that matured between scoring dates.

### Genotyping

The genetic information was collected from Illumina SoyNAM BeadChip SNP array specially designed for SoyNAM, comprising 5305 SNP markers selected from the sequencing of all 41 parental lines (Song *et al.* 2017). Missing loci were imputed using a hidden Markov model and removed markers with minor allele frequency below 0.05 using the R package NAM (Xavier *et al.* 2015). A total of 4240 SNPs were used for genomic analysis.

### Genetic merit

The genetic values were estimated as the best linear unbiased predictors (BLUP), as a random term of a mixed model. The mixed linear model was fitted with variance components based on restricted maximum likelihood (REML), computed using the R package lme4 (Bates *et al.* 2014). The linear model used to model genetic values:

y=μ+f(s)+Zu+Wg+e

Where the response variable y was modeled as a function of an intercept μ, spatial covariate f(s) based on a moving-average of neighbor plots as described by Lado *et al.* (2013) implemented in the functions NNscr/NNcov of the R package NAM (Xavier *et al.* 2015), a random effect Zu to capture the genetic effects of individual lines, namely the genetic effects, assumed to be normally distributed as $u∼N(0,\sigma^{2}{}_{u})$, a nuisance random effect Wg to capture the local environment effects, as normally distributed as $g∼N(0,\sigma^{2}{}_{g})$, and a vector eof residuals, normally distributed $e∼N(0,\sigma^{2}{}_{e})$. The inverse phenotypic variance was computed for each environment and used as observation weights to account for the heteroscedasticity among trials. Although the checks were not explicitly included in the genetic merit model, these were invaluable for the spatial correction of the field plot variation. Broad-sense heritability (H) was estimated from the REML variance components as:

$$H=\frac{\sigma^{2}{}_{u\;}}{\sigma^{2}{}_{u}+r^{-1}\sigma^{2}{}_{e}}.$$

Where r is the average number of replicates per entry. The reliability of the $j^{th}$ genotype ($H_{j}$) was used to deregress (Garrick *et al.* 2009) its corresponding BLUP ($u_{j}$) in order to obtain the genetic values in natural scale ($y_{j}=u_{j}/H_{j}$). This procedure of unshrink BLUPs precludes the downstream analyses to be performed upon a vector of phenotypes with heterogeneous degree of shrinkage, which may lead to biased results.

The narrow-sense heritability estimates ($h^{2}$) were based on the following SNP-BLUP model:

y=μ+Ma+ε

Where y correspond to the genetic values, modeled as a function of an intercept μ, matrix with SNP information and marker effects (Ma), and the vector of residuals (ε). Both marker effects and residuals were assumed to be normally distributed with variances $\sigma_{a}^{2}$ and $\sigma_{\varepsilon }^{2}$, respectively. The narrow-sense heritability was computed under two scenarios: 1) deploying all markers and 2) only with the markers found to be associated with yield components. The narrow-sense heritability was estimated as follows:

$$h^{2}=\frac{\sigma^{2}{}_{a}\times 2\sum_{j=1}^{J}p_{j}(1-p_{j})}{\sigma^{2}{}_{a}\times 2\sum_{j=1}^{J}p_{j}(1-p_{j})+\sigma^{2}{}_{\varepsilon }}.$$

### Polygenic epistasis

We performed a within-family variance component analysis to determine the amount of variability jointly explained by additive and additive-by-additive epistasis. For that, we fit a kernel-based model referred to as the G2A model (Zeng *et al.* 2005). Variance components were estimated using REML estimates (Misztal 2008). The analysis followed the linear model:

y=μ+ψ+ω+ε

$$\psi ∼N(0,K\sigma^{2}{}_{\psi })$$

$$\omega ∼N(0,Q\sigma^{2}{}_{\omega })$$

$$\varepsilon ∼N(0,I\sigma^{2}{}_{\varepsilon })$$

Where y correspond to the genetic values, modeled as a function of an intercept (μ), additive genetic values (ψ), additive epistatic value (ω), and the vector of residuals, (ε). The relationship matrices were built in accordance to Zeng *et al.* (2005) and Xu (2013). The additive genetic relationship matrix was obtained by the cross-product of the centralized marker matrix (M) with centralized trace, thus K=MM’α with $\alpha =n\times Tr{\left(MM’\right)}^{-1}$, and the additive epistatic relationship matrix was computed by the additive Hadamard product with centralized trace, thus Q=(MM’#MM’)α with a normalizing factor $\alpha =n\times Tr{\left(MM’\#MM’\right)}^{-1}$.

### Multivariate analysis of pleiotropy and stability

Multivariate analysis, namely genetic and additive genetic correlations, allows exploring the interaction between traits across years (pleiotropy) or within trait between years (stability or genotype-by-environment correlation). The genetic correlations within-family were obtained as the Pearson’s correlation between the BLUPs of yield and yield components for pleiotropy analysis, as well as the correlations of yield components from year to year for stability analysis. We estimated the additive genetic correlation between yield and yield components for pleiotropy analysis, and yield components across years for stability analysis, for each of the 40 families using a multivariate GBLUP model. The GBLUP model was fit with REML variance components. For the multivariate polygenic analysis, we fitted the following multi-trait model:

y=μ+ψ+ε

$$\psi ∼N(0,K⊗\Sigma_{\psi })$$

$$\varepsilon ∼N(0,I⊗\Sigma_{\varepsilon })$$

Where, under multivariate settings, $y=\{y_{1},y_{2},...,y_{k}\}$ correspond to the genetic merits, modeled as a function of their corresponding intercepts, $\mu =\{\mu_{1},\mu_{2},...,\mu_{k}\}$, the additive genetic values, $\psi =\{\psi_{1},\psi_{2},...,\psi_{k}\}$, and the residuals, $\varepsilon =\{\varepsilon_{1},\varepsilon_{2},...,\varepsilon_{k}\}$. With respect to the model variances, Kis the relationship matrix defined in the previous model, the additive covariances $\Sigma_{\psi }$ is a dense k×k matrix where the ij cell corresponds to the additive genetic covariance $\sigma_{\psi }\left(i,j\right)$ between $i^{th}$ and $j^{th}$ traits, and the residual covariance was assumed to be diagonal $\Sigma_{\varepsilon }=diag(\sigma^{2}{}_{\varepsilon 1},\sigma^{2}{}_{\varepsilon 2}\;,...,\sigma^{2}{}_{\varepsilon k})$. Additive genetic correlations were estimated from the covariance components as $\rho_{\psi }(i,j)=\sigma_{\psi }(i,j)/[\sigma_{\psi }(i)\sigma_{\psi }(j)]$. From the genetic correlations and heritabilities, the efficiency of indirect selection (Falconer and Mackay 1996) using $i^{th}$ trait to select the $j^{th}$ trait was estimated as $E=h_{j}{}^{-2}\;h_{i}\;{}^{2}\;\rho_{\psi }(i,j)$.

### Association studies

Since various signal detection strategies may capture different QTL (Yang *et al.* 2018), three complementary methodologies of genome-wide association studies were deployed in this study: Single marker analysis, implemented in the R package NAM (Xavier *et al.* 2015), whole-genome regression BayesCpi (Habier *et al.* 2011) implemented in the R package bWGR (Xavier *et al.* 2019), and random forest implemented in the R package ranger (Wright and Ziegler 2015). A brief description of the methods is provided below.

#### Mixed Linear Model (MLM):

This method of an association study is based on the likelihood ratio between a model containing the marker of interest (full model) and a model without the marker (reduced model). Both models include a polygenic term that accounts for the population structure. The statistical model that describes this association study is tailored to NAM populations (Xavier *et al.* 2015) and follows the linear model:

y=μ+Xβ+ψ+e

Where the genetic values (y) are modeled as a function of an intercept (μ), the matrix containing the interaction between the SNP information and family for the target marker under evaluation (X), the vector of marker effect within family $\beta ∼N(0,\sigma^{2}{}_{\beta })$, the vector of independent residuals, $\varepsilon ∼N(0,\sigma^{2}{}_{\varepsilon })$, and the polygenic term defined previously, $\psi ∼N(0,K\sigma^{2}{}_{\psi })$, which parametrizes the genetic covariance among individuals through the full-ranking genomic relationship matrix K. Bonferroni thresholds were utilized to account for multiple testing and mitigate false-positives, yielding a two-sided threshold of -log10(0.025/4240)=5.23. The association model was fit with REML variance components.

#### Whole-genome regression (WGR):

Designed primarily for prediction, WGR methods fit all markers at once. The prior distribution of marker effects follows a mixture of distributions to perform feature selection. The association statistics are based on the posterior probability of each marker to be included in the model, or “model frequency”. The model of choice, BayesCpi, assumes each marker has a probability π of being included in the model, where the parameter π is estimated in each MCMC iteration. Markers reached statistical significance if 1-π was smaller than a two-sided threshold of α=0.05, which translates into a threshold for the Manhattan plot of -log10(0.025)=1.6. The linear model that describes BayesCpi is the following:

y=μ+Ma+e

Where y correspond to the genetic values, modeled as a function of an intercept (μ), the matrix containing the all SNP information (M) and the vector of all marker effects jointly estimated (a), which followed a mixture of distributions, having probability π of having null effect and probability 1−π or being normally distributed as $N(0,\sigma^{2}{}_{\beta })$, and the vector of independent residuals, $\varepsilon ∼N(0,\sigma^{2}{}_{\varepsilon })$. The marker and residual variances were assumed to follow an inverse scaled chi-squared distribution, $\sigma^{2}{}_{\beta }∼\chi^{2}(S_{\beta },\nu_{0})$and $\sigma^{2}{}_{\varepsilon }∼\chi^{2}(S_{\varepsilon },\nu_{0})$, assuming $\nu_{0}=5$ prior degrees of freedom and shape parameters computed assuming prior heritability of 0.5 (Pérez and de Los Campos 2014), thus $S_{\beta }=0.5\;\sigma^{2}{}_{y}\;MSx^{-1}{\left(1-\pi \right)}^{-1}$and $S_{\varepsilon }=0.5\;\sigma^{2}{}_{y}$. The model was fit with 20000 MCMC iterations, discarding the initial 2000 iterations, and no thinning, such that the posterior means were computed by averaging 18000 MCMC iterations.

#### Random forest regression (RFR):

Random forest is a non-parametric regression derived from the bootstrapping aggregation of decision trees built from subsets of data and parameters. The association statistics of RFR is based on feature importance (Botta *et al.*, 2014). The forest was grown with 10000 decision trees. The trees were built having as starting point $\sqrt{m}=65$ SNPs sampled at random with replacement. The metric of variable importance was the ‘impurity’ index, which is a measure of the out-of-bad explained variance. Because there is no objective way of defining an association threshold for significant SNPs, we estimated the global empirical threshold (Doerge and Churchill 1996) based on 1000 permutations (α=0.05), thus making no assumptions about the distribution of the associations.

### Cross-validation studies

Cross-validations were performed for each yield component. Due to the known population structure of the SoyNAM, three types of cross-validations were performed: (1) within-family, (2) across-family, and (3) leave-family-out. Within- and across-family validations were performed as fivefold cross-validation, randomly selecting 80% of the data as a calibration set, and using the remaining 20% as a prediction target. The sampling and prediction procedure is repeated 25 times. Leave-family-out validation use 39 families to predict the family left out, and the procedure is performed to all 40 families. The prediction statistic is the predictive ability (PA), as the correlation between predicted and observed values.

The cross-validation was performed using the functions *emCV* of the R package bWGR (Xavier *et al.* 2019). In accordance with the genomic prediction benchmark proposed by Daetwyler *et al.* (2013), two statistical models evaluated in this study were GBLUP (VanRaden 2008) and BayesB (Meuwissen *et al.* 2001). The GBLUP model was fitted as a ridge regression with REML variance components, and the BayesB assumes that markers effects follow a mixture of distribution, where the $j^{th}$ marker had probability π=0.95 of having null effect and probability 1−π of being normally distributed as $N(0,\sigma^{2}{}_{\beta j})$, variances were assumed to follow an inverse scaled chi-squared distribution, $\sigma^{2}{}_{\beta j}∼\chi^{2}(S_{\beta },\nu_{0})$and $\sigma^{2}{}_{\varepsilon }∼\chi^{2}(S_{\varepsilon },\nu_{0})$, assuming $\nu_{0}=10$ prior degrees of freedom and shape parameters computed as $S_{\beta }=0.5\;\sigma^{2}{}_{y}\;MSx^{-1}$and $S_{\varepsilon }=0.5\;\sigma^{2}{}_{y}$.

### Data availability

All phenotypic and genotypic data are available in the R package SoyNAM available on CRAN. To access the data, install the SoyNAM package (CRAN.R-project.org/package=SoyNAM), then load the Indiana dataset with the following command in R: data(soyin, package=′SoyNAM′).

## Results

**Figure 1:**
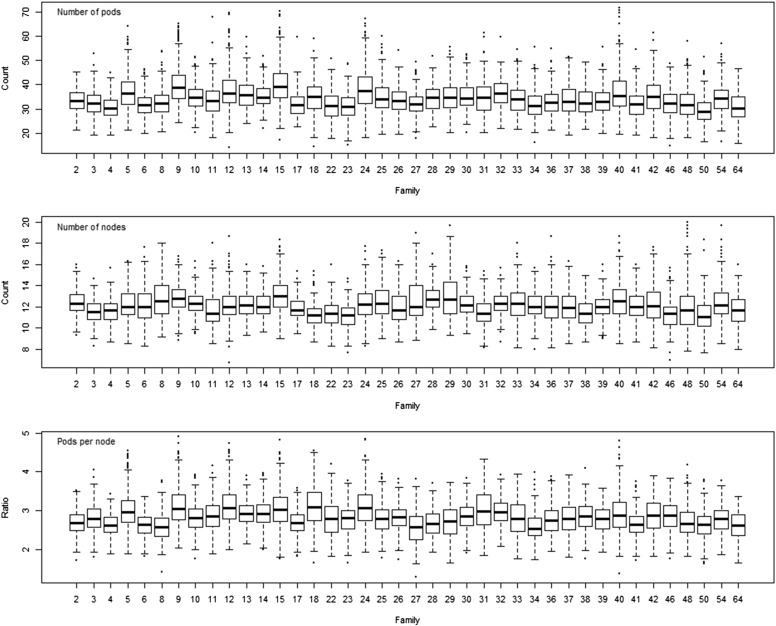
Phenotypic distribution of the pod number (top), node number (center) and pods per node (bottom). Families had elite (2-23), diverse (2-39) and exotic (40-64) genetic background.

The SoyNAM provided reasonable variation for the three yield components. The phenotypic distributions of the yield components for each of the SoyNAM families is presented in Figure 1. The mean and standard deviation across families is provided in Table 1, alongside the broad- and narrow-sense heritability estimated across families.

**Table 1:** Trait distribution (mean and standard deviation) and genetic metrics: broad-sense heritability (H), narrow-sense heritability ($h^{2}$) estimated using all SNPs and the subset of significant SNPs

Trait	Mean	Std. Dev.	H	$h^{2}$(all SNPs)	$h^{2}$ (QTL SNPs)
Nodes	12.085	1.090	0.352	0.159	0.069
Pods	34.046	4.955	0.361	0.110	0.095
P/N	2.819	2.819	0.301	0.064	0.142
Yield	66.557	14.345	0.334	0.280	0.093

The broad-sense heritability of the number of pods and nodes was slightly higher than the broad-sense heritability of yield, however, the narrow-sense heritability of yield was almost twice as large and the number of nodes, and almost three times higher than the narrow-sense heritability of the number of pods. The narrow-sense heritability estimated from the 18 markers found associated with yield components recovered almost entirely the narrow-sense heritability of the number of pods, but just a third of the heritability of the number of nodes and grain yield. And, surprisingly, the narrow-sense heritability of the ratio of pods per node was higher when only the significant markers were used.

### Association analysis

**Figure 2:**
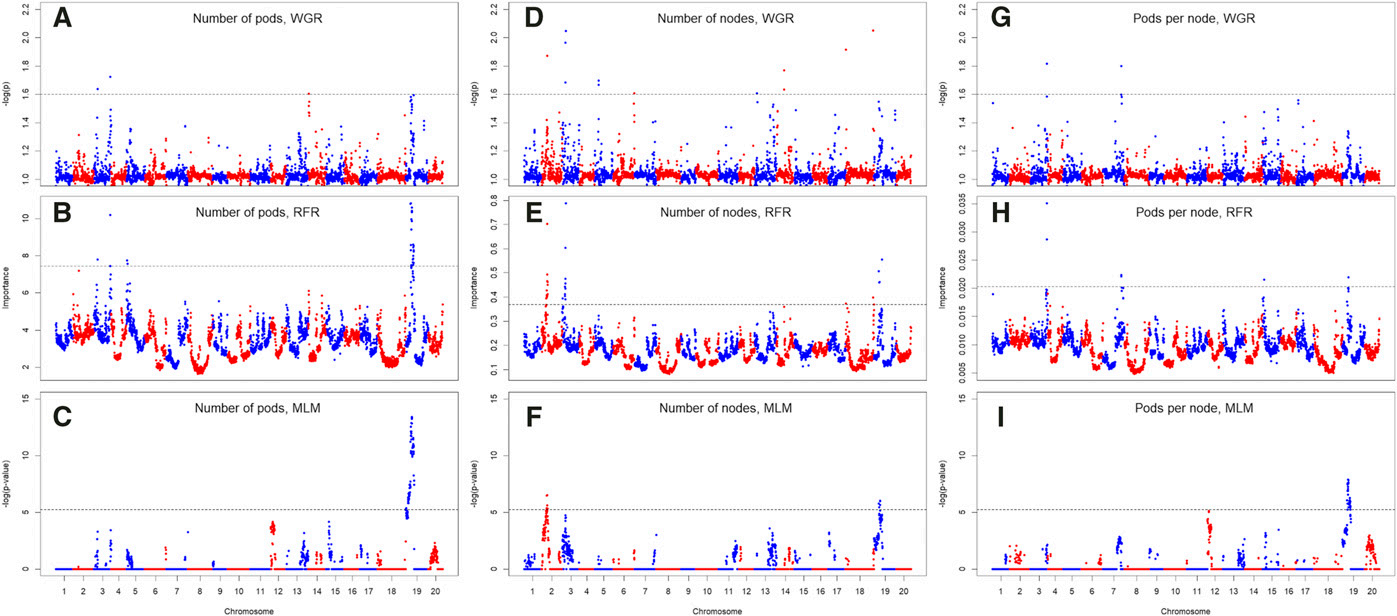
Genome-wide association studies of pod number (A,B,C), node number (D,E,F) and pods per node (G,H,I), performed through three methodologies: WGR whole-genome regression (A,D,G), RFR random forest regression (B,E,H), and MLM mixed linear model (C,F,I). RFR significance is defined by permutation threshold; MLM significance is adjusted for multiple testing with Bonferroni threshold; WGR does not require adjustment for multiple testing.

The genome-wide screening for segments associated to yield components is presented in Figure 2. Regions associated with the number of pods were located in chromosomes 3, 5, 14 and 19; significant associations for node number were observed in chromosomes 2, 3, 5, 6, 14, 18 and 19; and regions associated with pods per node were detected in chromosomes 3, 7, 12 and 19. The summary of the associated regions is presented in Table 2, alongside the impact of each significant marker on the yield components, grain yield and days to maturity. With the exception of the association between the marker Gm02_6396340 and the number of nodes, our study did not find any other consensus QTL detected by all three association methods for any of the yield components. All three yield components had significant associations in chromosomes 3 and 19, and the marker Gm19_1587494 was associated with all three traits. From the associated markers, Gm13_14346156 had the highest impact on grain yield, potentially increasing yield as much as 0.6 bushels per acre.

**Table 2:** Summary of association studies: SNP at the peak of each QTL; corresponding trait and method from which the QTL was identified, and the least squared effect of the SNP for each yield components, yield and days to maturity. Negative values indicate the desirable allele is inherited from founder parents

SNP	GWAS (Figure 2)	Number of pods	Number of node	Ratio pods ped node	Yield (bu/ac)	Days to Maturity
Gm02_6396340	B,D,E,F	−0.26	−0.41	−0.03	−0.47	−0.16
Gm03_2182974	A,D,E	−0.25	−0.38	−0.03	−0.18	−0.33
Gm03_46533591	A,B,G,H	−0.32	−0.12	−0.34	0.07	0.02
Gm05_914933	B	−0.20	−0.17	−0.10	0.15	0.02
Gm05_3661638	B,C	0.13	0.25	−0.05	−0.23	0.30
Gm06_47199506	D	0.10	0.21	−0.05	0.22	0.06
Gm07_7868756	G,H	−0.02	0.18	−0.18	0.05	−0.03
Gm12_2838455	I	0.07	0.15	−0.04	−0.09	0.08
Gm13_14346156	D	0.19	0.26	0.05	0.62	0.07
Gm14_743883	A	0.23	0.16	0.18	0.18	0.13
Gm14_917668	B	0.23	0.19	0.15	0.11	0.22
Gm14_2322106	D	0.17	0.27	−0.01	0.40	0.32
Gm15_5446785	H	−0.09	0.14	−0.23	−0.24	−0.02
Gm18_2357823	D,E	−0.11	−0.24	0.05	−0.27	−0.02
Gm18_57370051	D,E	0.23	0.28	0.08	0.14	0.22
Gm19_1496625	B,E,I	−0.43	−0.38	−0.28	−0.34	0.02
Gm19_1587494	B,C,F,H,I	−0.43	−0.33	−0.31	−0.15	0.09
Gm19_1991181	E	−0.36	−0.34	−0.21	−0.15	0.10

### Polygenic architecture

**Figure 3:**
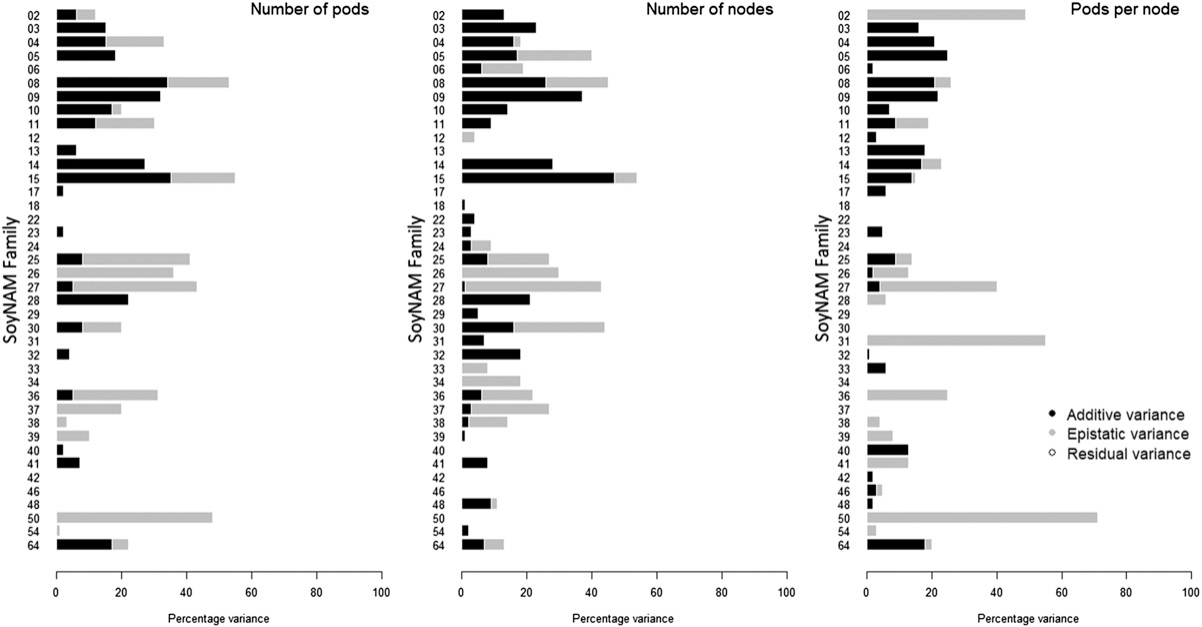
Barplot of the proportion of variance explained by different genetic components of pod number (left), node number (center) and pods per node (right) by family. Additive (black), epistatic (gray) and residual (white) variances. Families had elite (2-23), diverse (24-39) and exotic (40-64) genetic background.

**Figure 4:**
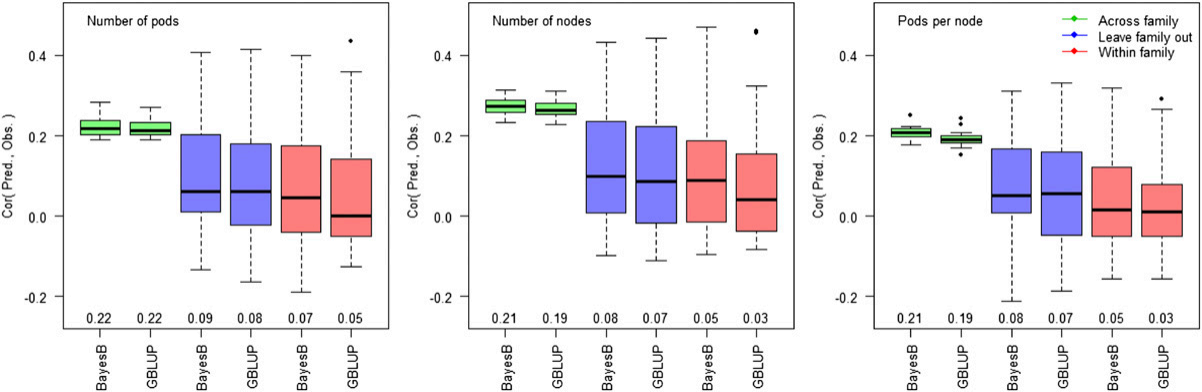
Boxplot of predictive ability of pod number (left), node number (center) and pods per node (right), where two prediction models (BayesB and GBLUP) tested three cross-validations strategies: across-family (green), leave-family-out (blue) and within-family (red). The three cross-validations schemes provide an insight on across-family selection (across family), prediction and selection of individuals from unobserved family (leave family out), and within family selection that capture only QTL segregating in the family under evaluation (within family).

The proportion of variance explained by additivity and epistasis for individual families is presented in Figure 3. The additive fraction of the genetic variance computed using G2A kernels is comparable to the narrow-sense heritability estimated across families (Table 1). All three yield components presented similar average polygenic architecture, having the additive and epistatic components ranging from 0 to approximately 50%, but the estimates where highly variable from family to family. The additive component averaged 7.46%, 9.03%, and 6.18%; the epistatic component averaged 7.92%, 7.02%, and 7.77%, and the total genomic heritability (additive + epistatic components) averaged 15.38%, 16.05%, and 13.95% for the number of pods, nodes, and pods per node, respectively. Many families provided near-zero genetic control for yield components, in agreement with the low within-family predictive ability (Figure 4).

### Prediction analysis

The outcome of the prediction analysis is presented in Figure 4. Predictions within-family provided lower correlations than leave-family-out, and across-family predictions yielded the most predictive scenario. All three yield components had similar heritabilities (Table 1) and, consequently, similar prediction accuracies. For the different cross-validation scenarios, correlations around 0.05, 0.08 and 0.21 were observed for predictions within-family, leave-family-out, and across-families, respectively. BayesB provided a slightly higher predictive ability than GBLUP across cross-validation scenarios, providing an increase in predictability of as much as 0.02. However, the differences in predictive ability were negligible, in agreement with previous results (Xavier *et al.* 2016). The slightly advantageous performance of BayesB suggests that some QTL contribute to the prediction of yield components, but a polygenic model captures most of the genomic signal.

### Genetic correlations and indirect selection

**Figure 5:**
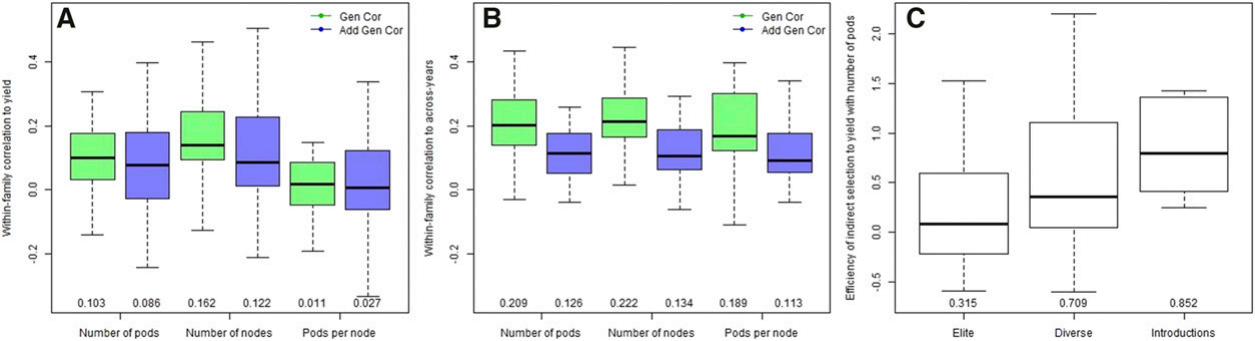
Pleiotropy yield and yield component (A), genotype-by-environment correlation (B) and efficiency of indirect selection (C). Boxplot displaying the dispersion of within-family genetic and additive-genetic correlation between yield components and grain yield (A); the within-family genetic and additive genetic genotype-by-environment correlation (B), where more means more stable across years; the efficiency of indirect selection to yield using pods, breakdown by germplasm background (C).

The within-family genetic and additive genetic correlations between yield components and yield, as well as yield components stability, are presented in Figure 5. Whereas the average correlations between yield components and yield are relatively small (Figure 5A), there is a large variation from family to family, which indicates that some families could benefit from the selection of yield components. From the three yield components, the number of pods was the only trait with the efficiency of indirect selection that departed from zero (data not presented), so we broke down the efficiency of indirect selection based on pod counts by the genetic background of the SoyNAM founder (Figure 5C). Families with non-elite genetic backgrounds are more likely to benefit, and the indirect selection based on pods was more effective than on yield itself in 10 families (E>1).

## Discussion

The dissection of yield components using multiple quantitative genetic approaches using genomic information provides an insight on how such traits can be utilized for breeding purposes. For that, we performed a wide range on analysis, including checking the heritability in broad- and narrow-sense, whether there were major genes involved, whether these genes are captured by different approaches of association analysis, whether the genetic control is influenced by epistatic factors, the trait stability across years, and genomic predictive ability in different settings, different models, within and across families. The collective interpretation of these analysis contributes to the construct the big picture of the genetic architecture of these traits.

### Brief overview of the architecture

The number of pods and nodes, as the ratio pods per node, are key yield components in soybean (Herbert and Litchfield 1982) reported to be yield drivers (Kahlon and Board 2012, Suhre *et al.* 2014). Understanding how such traits work may provide insight into better strategies to increase yield and yield stability (Xavier *et al.* 2017a). In soybeans, we found that these yield components have low heritability, both in the broad and narrow sense, and have partially oligogenic architecture, where the genomic control is jointly explained by a set of QTL and polygenic terms (Figures 2 and 3). In addition, within-family analysis indicates that some populations display more epistatic than additive control under the polygenic model (Figure 3), whereas other families presented no genetic control whatsoever.

### QTL

Successful mapping of markers associated with complex traits relies on the size and variability of the mapping population. Our study was conducted on the SoyNAM, a large population designed to optimize power and resolution. Yet, only a small number of QTL were detected. Previous mapping studies on yield components have relied on non-experimental panels with a highly diverse genetic background. The studies of Hao *et al.* (2012), Hu *et al.* (2014), Zhang *et al.* (2015) and Fang *et al.* (2017) assessed 191, 113, 219 and 809 genotypes, respectively, including landraces and wild accessions. Among the studies on diverse backgrounds, Fang *et al.* (2017) found a QTL for pod and node numbers in close proximity to our QTL peak on chromosome 06, marker Gm06_47199506. The pod number QTL detected by Hu *et al.* (2014) were located in chromosomes 3, 5 and 6, in overlapping regions to signals Gm03_2382974, Gm03_46533591, Gm05_914933, Gm05_3661638, Gm06_47199506, and Gm07_7868756.

The significant markers found from this study do not overlap with the signals found for grain yield (Diers *et al.* 2018) and yield stability (Xavier *et al.* 2018) in the SoyNAM population. However, markers Gm02_639640, Gm07_7868756 and Gm12_2838455 are in close proximity to seed size QTL reported by Diers *et al.* (2018). Two markers, Gm19_1587494 and Gm18_57370051, were found to be associated with important traits from previous studies. The marker Gm19_1587494 was also found to be the key association to canopy coverage (Xavier *et al.* 2017b), which means that canopy coverage could be associated with the three yield components. The marker Gm18_57370051 is linked to the stem termination gene Dt2 (Bernard *et al.* 1972), which has been previously detecting in NAM families by Ping *et al.* (2014). In previous studies, Hao *et al.* (2011) and Fang *et al.* (2017) found that Dt2 is an influential gene on the number of pods and nodes. The Dt2 gene is also believed to have played a role in the soybean domestication (Sedivy *et al.* 2017).

The markers that were found to be associated to yield components in this study had little to no impact in maturity, which can be a major limiting factor to their use in breeding as most QTL that improve yield often increase the number of days to maturity (Table 2). However, the QTL peaks also had a limited impact on grain yield across family, with effects ranging from -0.46 to 0.62 bu/ac.

It is important to point out that Table 2 presents an average effect of allele substitution for simplicity. However, two association methods deployed in this study do not directly estimate the allele effects: The MLM utilized in this study computes the significance from within-family effects, hence capturing signal in different linkage phases between marker and QTL. The RFR also does not necessarily provide an allele effect, instead it computes recursive decision trees that would capture QTL with additive, dominant or epistatic effect. Therefore, the intend of this study was mostly focused on tracking which markers are likely associated to the yield components rather than inferring from which parent the desirable alleles are inherited from.

### Genomic selection

Markers are informative in two levels for genomic predictions: they can inform the relationship and detect markers linked to, or under linkage disequilibrium with, the quantitative trait loci (Habier *et al.* 2007). Within-family predictive ability solely relies on the linkage disequilibrium (LD) between markers and QTL, as the relationship among individuals is constant. The predictions of families not included in the training set (leave-family-out) can yield mixed results since the training set often holds families with shared ancestry. Of course, the controlled ancestry is a key property of NAM populations since all families share a common parent and, therefore, the outcome predictive ability is higher than the non-experimental population where neither parent has offspring in the calibration set. Predictions performed across family are presumably the most likely to be accurate, as they capture relationships among families and disequilibrium between markers and QTL.

Figure 4 depicts well the expected predictive ability, as within-family predictions hold a high degree of uncertainty, with correlations averaging from 0.064 across yield components, followed by leave-family-out predictions, with an average correlation of 0.092, and the most predictive was across-family predictions, with average correlations above 0.224. Predictive abilities computed from leave-family-out and within-family can be penalized from the fact that some families presented had near-zero heritability and hence no variation for yield components. Within-family predictions may be further penalized due to the small population size to calibrate the genomic models. However, across family predictions are relatively more accurate as those capture both relationship and LD information, and the lower dispersion of the predictions can be attributed to the fact that the prediction model is large, containing a large number of full- and half-siblings. Results from the enhancement in predictive ability due to the joint availability of LD and relationship information have been previously presented from a theoretical standpoint by Habier *et al.* (2013) and Schopp *et al.* (2017), and similar results in real data were reported by Ogut *et al.* (2015) in the maize NAM population. In hybrid maize study, Lehermeier *et al.* (2014) claimed 375 half-siblings to provide the same predictive ability of 50 full-siblings, but emphasized the degree of relatedness among families would also play a key role in the predictive ability.

Prediction accuracies estimated across families can also have a misleading interpretation, as these are subject to the Simpson paradox (Chipman and Braun 2017), where the model is able to detect large differences across families, but the predicted families may display negative correlation within-family. Such limitations could be addressed if the cross-validations across-family were performed sampling 20% of individuals from each family and training with the remaining set comprising all families, then estimating the average within-family predictions. However, across-family validations have two advantages: (1) these indicate the predictive potential of selections performed across populations and (2) provide results that can be more easily compared to other literature reports, as most studies performed cross-validations disregarding within-population studies.

The difference in predictive ability between GBLUP and BayesB, which translated into an average improvement of 0.02 going from GBLUP to BayesB, is due the larger flexibility the BayesB model, which is more likely to capture large effects and perform variable selection (Meuwissen *et al.* 2001, Habier *et al.* 2011, Pérez and de los Campos 2014, Xavier *et al.* 2016). Having a comparison between GBLUP and BayesB can provide an insight into the genetic architecture of the trait under evaluation (Daetwyler *et al.* 2013). In this study, we expected BayesB to outperform GBLUP since we uncover a partially oligogenic architecture from the association analysis, but a key piece of information that the genomic prediction analysis provides is discrepancy between GBLUP and BayesB, which inform the degree to which the genetic architecture of the traits under evaluation depart from a polygenic architecture.

Note that the advantage provided by changing the model from GBLUP to BayesB is nowhere comparable to the difference in predictability between cross-validation methods (*i.e.*, within-family, leave-family-out, and across-family). The reason why this phenomenon occurs is that different methods may improve how well the model detects the genetic architecture, but different types of cross-validation provide different information. Thus, gains associated to the choice of a prior are often considered negligible in comparison to increases in population size, better experimental practices, or more representative calibration sets (de los Campos *et al.* 2013, Xavier *et al.* 2016). A possible way of capturing more information for genomic prediction is the explicit modeling of other sources of genetic information, such as dominance and epistasis (Xu 2013). As presented in this study, yield components in some populations have a greater influence of epistasis than the additive background and, on average, the within-family variance decomposition indicates that additive genetics explains as much of the yield components phenotypes as epistasis (Figure 3).

### Stability and plasticity

When assessing genotype-by-environment, the total genetic correlation was larger the additive genetic correlation for all yield components (Figure 5B), approximately ranging from 0 to 0.4, whereas the additive genetic correlations ranged from 0 to 0.25. The discrepancy between genetic and additive genetic correlations is attributed to the genetic control due to QTL and non-additive polygenic genetic background.

For the families with near-zero genotype-by-environment correlation, performing selections with a single year of data may not reflect into observable genetic gains in the coming years, and that collecting data from more environments may not necessarily increase the predictive ability of the yield components. Particularly for yield components, low genotype-by-environment correlations is not necessarily bad since the soybean yield plasticity relies on reallocating resources among yield components, which serves as a physiological response to mitigate yield losses under stress (Board and Tan 1995, Board *et al.* 1997, Pedersen and Lauer 2004, Zhang *et al.* 2004). Whereas yield components are mainly responsible for the yield formations, these are not necessarily the best linear yield predictors (Board and Modali 2005). For example, Board and Harville (1993) showed that the number of pods serves as the mechanism by which seed production increases in response to greater light interception.

Our previous study (Xavier *et al.* 2017a) assessed the association among soybean agronomic traits and yield components in the SoyNAM population-based on undirected graphical models. The graphical models depicted genetic and environmental interdependence among yield components. That means that interactions among yield components occur due to genetic forces as well as a response to environmental stimuli and agronomic practices. Such a phenomenon is also described in a summary of agronomic studies on soybean yield components authored by Board and Kahlon (2011). The interactions among yield components play a key role in the redistribution of resources and yield stability (Ball *et al.* 2000). It is possible that breeding any given yield component toward extreme values may result in a compromised ability of soybeans to compensate yield under stress (Malausa *et al.* 2005).

### Yield increases

From the standpoint of trait decomposition, the breaking down of grain yield into pods and nodes does not seem to be an effective approach since there is no strong evidence that these yield components are more heritable than yield (Table 1) or strong genetic correlation to yield (Figure 5A) that would justify the selection based on yield components. With the exception of a few families, yield components are not good proxies for grain yield (Figure 5C). It is possible that the genetic architecture of the yield components under evaluation is just as complex as grain yield itself, not justifying predicting yield components instead of yield *per se*.

In our previous genomic prediction study (Xavier *et al.* 2016), we assessed how a variety of different genomic prediction models would predict the agronomic traits and yield components under the following scenario: within year and across-population. Even though that study did not provide in-depth insight into the genetic architecture of yield components, it was found that genomic prediction models that can jointly account for large effect QTL and epistasis were advantageous over simpler prediction approaches. That study also found that predicting yield is easier than predicting yield components. Those results were further confirmed by the current study, where we assessed the architecture of yield components with more data and under different approaches.

### Phenotyping

A major challenge of working with yield components is the data collection as the counting is highly subjective to human error, lowering the trait heritability and affecting the signal detection in downstream analysis. As deep learning methods for computer vision become increasingly population for phenotyping morphological traits (Singh *et al.* 2018), the current limitations with data collection could be addressed by an automated high-throughput phenotyping instead of human counts, that would likely increase both accuracy and scalability of the process. A recent study by Zhang *et al.* (2019) provides a procedure using computer vision for counting soybean pod under experimental settings that would address the phenotypic limitation of this study. Similarly, Uzal *et al.* (2018) and Li *et al.* (2019) recently proposed an imagery system for counting seeds directly from images of soybean pods, yet another yield component limited by the challenging phenotyping. Technologies that enable better, faster and cheaper data collection remain a key limiting factor for the research in yield components.
